# Killer Whale Depredation and Associated Costs to Alaskan Sablefish, Pacific Halibut and Greenland Turbot Longliners

**DOI:** 10.1371/journal.pone.0088906

**Published:** 2014-02-18

**Authors:** Megan J. Peterson, Franz Mueter, Keith Criddle, Alan C. Haynie

**Affiliations:** 1 School of Fisheries and Ocean Sciences, University of Alaska Fairbanks, Juneau, Alaska, United States of America; 2 Resource Ecology and Fisheries Management Division, Alaska Fisheries Science Center, Seattle, Washington, United States of America; Institut Maurice-Lamontagne, Canada

## Abstract

Killer whale (*Orcinus orca*) depredation (whales stealing or damaging fish caught on fishing gear) adversely impacts demersal longline fisheries for sablefish (*Anoplopoma fimbria*), Pacific halibut (*Hippoglossus stenolepis*) and Greenland turbot (*Reinhardtius hippoglossoides*) in the Bering Sea, Aleutian Islands and Western Gulf of Alaska. These interactions increase direct costs and opportunity costs associated with catching fish and reduce the profitability of longline fishing in western Alaska. This study synthesizes National Marine Fisheries Service observer data, National Marine Fisheries Service sablefish longline survey and fishermen-collected depredation data to: 1) estimate the frequency of killer whale depredation on longline fisheries in Alaska; 2) estimate depredation-related catch per unit effort reductions; and 3) assess direct costs and opportunity costs incurred by longliners in western Alaska as a result of killer whale interactions. The percentage of commercial fishery sets affected by killer whales was highest in the Bering Sea fisheries for: sablefish (21.4%), Greenland turbot (9.9%), and Pacific halibut (6.9%). Average catch per unit effort reductions on depredated sets ranged from 35.1–69.3% for the observed longline fleet in all three management areas from 1998–2012 (p<0.001). To compensate for depredation, fishermen set additional gear to catch the same amount of fish, and this increased fuel costs by an additional 82% per depredated set (average $433 additional fuel per depredated set). In a separate analysis with six longline vessels in 2011and 2012, killer whale depredation avoidance measures resulted in an average additional cost of $494 per depredated vessel-day for fuel and crew food. Opportunity costs of time lost by fishermen averaged $522 per additional vessel-day on the grounds. This assessment of killer whale depredation costs represents the most extensive economic evaluation of this issue in Alaska to date and will help longline fishermen and managers consider the costs and benefits of depredation avoidance and alternative policy solutions.

## Introduction

**Figure 1:**
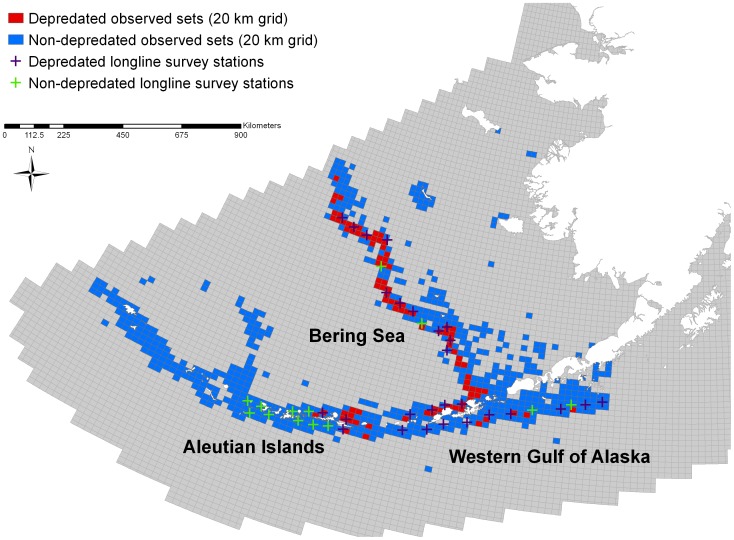
NMFS Longline Survey Stations and Observer Sets Sampled 1998–2012. Map of western Alaska regions with depredated and non-depredated sets based on NMFS observer data and depredated and non-depredated stations based on the NMFS annual sablefish longline survey. Observer data are aggregated by 20 km grids for confidentiality.

Killer whale (*Orcinus orca*) depredation occurs when killer whales remove or damage hooked fish as the gear is being retrieved [1], [2]. While depredation by killer whales occurs in all ocean basins [2], [3], the issue of killer whale depredation is particularly significant in western Alaska where high-value longline fisheries overlap with some of the greatest densities of “fish-eating” or resident killer whales in the world [4], [5]. Killer whale depredation is most problematic in the Bering Sea (BS), Aleutian Islands (AI) and Western Gulf of Alaska (WGOA) fisheries management areas (Figure 1) but also occurs in Prince William Sound [5]–[8]. These regions support major demersal longline fisheries for sablefish (*Anoplopoma fimbria*), Pacific halibut (*Hippoglossus stenolepis*) and Greenland turbot (*Reinhardtius hippoglossoides*), which are the primary fisheries affected by killer whale depredation in Alaskan waters [5], [8]. Killer whale depredation is less problematic in the Central and Eastern Gulf of Alaska and Southeast Alaska, where sperm whale depredation is the primary toothed whale interaction affecting demersal longline fisheries [9].

Killer whales can remove up to 30% of overall catches and up to 100% of catches on individual sets from longline fisheries targeting species including sablefish, Greenland turbot and Pacific halibut in the North Pacific and Patagonian toothfish (*Dissostichus eleginoides*) in the Southern Ocean[5], [10]–[13]. In addition to revenue reduction from lost catches, fishing fleets incur increased costs due to reduced catch per unit effort (CPUE) and changes in fishing practices to avoid depredating killer whales [7], [14], [15]. In a study evaluating changing fishing practices to minimize economic losses when encountering depredating killer whales, fishermen reported two primary methods to avoid killer whales: dropping their gear back down to “wait the whales out” and steaming to a different fishing site to “outrun the whales.” In this same study, fishermen operating primarily in the BS, AI and WGOA reported average wait times greater than 13 hours (hrs) and steaming on average at least 25 nautical miles (nm) to avoid depredating whales [7]. These mitigating measures lead to extended trip durations, increased travel distances, and lengthened gear soak times. These deviations from preferred fishing practices increase fuel consumption, bait costs, and crew expenditures and reduce opportunities for the vessel and crew to engage in additional fisheries or other income-generating opportunities.

The frequency of reported *odontocete* (toothed whale) interactions with longline fisheries increased globally from 1960 to 2010 [2]. This increase has been attributed to the modernization and geographic expansion of longline fishing during the mid- to late-twentieth century and the establishment of international conservation agreements to protect marine mammals, such as the International Convention on the Regulation of Whaling, and legislation enacted by individual nations, such as the U.S. Marine Mammal Protection Act and the U.S. Endangered Species Act [2]. There is a growing body of scientific literature investigating depredation frequency and catch removals by toothed whales; however, there are few studies examining the economic impacts of whale depredation on longline fleets.

Reported estimates indicate that toothed whale depredation can be costly for longline fleets. For example, based on the market price of Patagonian toothfish and predicted catch losses, it was estimated that between 2003 and 2008, killer whales and sperm whales were responsible for annual losses ranging from $800,000–$2 million per year in the Crozet Islands Exclusive Economic Zone (EEZ) [13], [16]. In dockside interviews conducted in Dutch Harbor, Alaska, during the 1988 fishing season (representing 147 days at sea), commercial longline skippers reported that they lost an average of $2,300 per day due to killer whale depredation (based on lost catch only and a 20% depredation rate) [14]. A more recent study of false killer whale (*Psuedorca crassidien*) and pilot whale (*Globicephala macrorhynchus*) interactions with swordfish (*Xiphias gladius*) and tuna (*Thunnus spp.*) longline fisheries off the Hawaiian Islands used lost catch and additional daily fuel and labor costs to estimate that tuna and swordfish fisheries could be losing $2,565 to $4,596 respectively per depredated set due to whale interactions [17].

Management and harvesting practices in the sablefish, Pacific halibut, and Greenland turbot longline fisheries have evolved over the last 20 years. The Pacific halibut and sablefish fisheries were converted to Individual Fishing Quota (IFQ) systems in 1995 to address problems associated with the “derby-style” short season and excess fleet capacity [7], [18]–[20]. As a result of that transition, Pacific halibut and sablefish longline fisheries are typically open from March to November [21]. The IFQ entitles fishermen to an exclusive share of the Total Allowable Catch assigned to their vessel size category in a particular geographic region for sablefish or Pacific halibut. Fishermen may hold IFQ for both species in multiple regions. Most sablefish quota is harvested in May, whereas most halibut quota is harvested in June [22]. The Greenland turbot fishery opens in May, but the majority of longline harvest occurs between June and August to avoid killer whale depredation [23]. In lengthening the active fishing season, IFQs may have had the unexpected consequence of inducing increased levels of depredation [7].

The goals of this study were threefold: 1) to estimate the percentage of commercial fishing sets impacted by killer whale depredation in western Alaska, 2) to estimate the effect of killer whale depredation on CPUEs, and 3) to estimate depredation-associated increases in operation and opportunity costs incurred by the Alaskan sablefish, Greenland turbot and Pacific halibut longline fleets operating in the BS, AI or WGOA. This evaluation of killer whale depredation on commercial fisheries serves as a first step towards understanding the economic impacts of killer whale depredation and how these costs may be factored into future management and depredation mitigation strategies.

## Materials and Methods

The goal of these analyses was to examine the frequency of depredation occurrence, CPUE reductions, direct costs, and opportunity costs for fishermen. CPUE-reduction analyses relied on National Marine Fisheries Service (NMFS) observer data; depredation-occurrence analyses relied on data from fishermen respondents and NMFS observer data. Cost estimates relied on a combination of the CPUE analyses and information provided by fishermen respondents. These methods are detailed in the following sections.

### 2.1 Killer whale depredation occurrence

The frequency of killer whale depredation was estimated using NMFS observer data from 1998 to 2012 for the BS, AI and WGOA and depredation data collected by fishermen during the 2011 and 2012 fishing seasons. Additional depredation frequency data were included from previous studies using NMFS sablefish longline survey data (depredation recorded per skate or string of 45 hooks)[5] and written surveys conducted with longline fishermen (depredation recorded per set)[7]. In federal waters off Alaska, observers were required to monitor approximately one third of fishing operations of the Alaskan longline fleet for vessels over 60 ft. in length and to monitor all fishing operations for vessels over 125 ft. Observers monitored and recorded species-specific catch data, fishing location information and general gear performance. A total of 228,538 sets were sampled in the BS, AI and WGOA. Each set was assigned a performance code (‘no problem,’ ‘considerable killer whale predation,’ ‘gear entanglement,’ ‘crab pot in set,’ etc.). Only sets with ‘no problem’ or ‘considerable killer whale predation’ as performance codes (227,785 sets) were included in the analysis. Per instructions in the NMFS observer manual, observers noted if there was considerable killer whale depredation based on visual evidence of killer whales interacting with the gear and feeding on catch [24].

The basic unit of gear for the NMFS observer data analysis was a set. Each set consists of one string of hooks (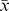 = 12,165 hooks per set) fished end to end by an observed longline vessel. Following NMFS guidelines, the target species of each set was assigned based on whichever groundfish species was most prevalent in the set [25]. This naïve rule is unable to differentiate between a halibut fishing trip that, in one set, caught more of some other species and a fishing trip for that other species. Consequently, this NMFS rule could result in a biased estimate of the number of sets by longline fishermen in the sablefish, Pacific halibut, and Greenland turbot fisheries. Nevertheless and in keeping with NMFS practices, the analyses described below are based on sets predominated by sablefish (5,716 sets, average bottom depth 320 m), Greenland turbot (5,915 sets, average bottom depth 336 m), and Pacific halibut (4,118 sets, average bottom depth 153 m). CPUE by species was estimated by dividing the total species weight (kg) per set by the total number of hooks per set. The proportion of sets depredated by killer whales was calculated separately for sets with sablefish, Pacific halibut or Greenland turbot as the assigned target species.

Fishermen operating in western Alaska during the 2011 and 2012 fishing seasons also collected depredation frequency data. Participants in this study were selected based on semi-directed interviews conducted with approximately 70 longline fishermen in Alaska from 2010–2011 [7]. During the interview process, six key informants (respondents) were selected to collect depredation data on the fishing grounds throughout the 2011 and 2012 fishing seasons (March to November). Fishermen respondents were selected based on their long-term fishing experience, time spent on the fishing grounds, and willingness to participate. This purposive sampling method enables researchers to work with particularly knowledgeable fishermen, but it limits the theoretical basis for making larger, fleet-wide inferences [26], [27].

Respondents were asked to report basic vessel and crew information for the entire season and to complete a “depredation sheet” for every day that whale interactions occurred. On the daily depredation sheets, fishermen recorded: date; number of sets fished for the day; number of sets affected by whales; fishing location; minimum and maximum estimates for numbers of whales present; and the estimated percentage of catch taken. Fishermen respondents submitted the completed depredation sheets via mail at the end of 2011 and 2012 fishing seasons. Vessels from three size categories participated in the study: three catcher vessels less than or equal to 60 feet; one catcher vessel greater than 60 feet; and two catcher-processors. The total number of sets fished for a given vessel was calculated by multiplying the reported days fished by the reported average number of sets fished per day. Altogether, these six vessels fished for 262 fishing days or approximately 846 sets in the BS, AI, or WGOA areas where killer whale depredation is prevalent. The proportion of sets or days impacted by killer whale depredation was calculated by dividing the number of reported sets or days affected by killer whales by the reported total number of days or sets fished.

### 2.2 Observed fishery CPUE Reductions

A statistical modeling approach was used to evaluate CPUE reductions incurred by the longline fleet in western Alaska due to killer whale depredation. NMFS observer data from 1998 to 2012 was analyzed to compare CPUE between sets with and without significant killer whale depredation in each management area: BS, AI and WGOA. A Generalized Additive Modeling framework (GAM; as implemented in the R package ‘mgcv’) was used to model sablefish, turbot and halibut CPUE as a function of killer whale depredation and included additional non-parametric functions of potentially important covariates in each management area [28]–[30]. The response variable was log-transformed sablefish, turbot, or halibut CPUE. Explanatory variables considered included year, vessel, and killer whale depredation as categorical variables; and smooth functions of location (latitude, longitude) and bottom depth as continuous variables. Interaction terms such as an interaction between killer whale depredation and year or killer whale depredation and vessel, were also examined [31]. The maximum degrees of freedom for all smooth terms was restricted to 5 to limit the analysis to biologically reasonable relationships. The Akaike information criterion (AIC) was used to select the “best” model for each target fishery [30], [32]. CPUE reductions due to killer whale depredation were calculated using the model-estimated killer whale depredation coefficients(*kw*), which represent the average difference in log(CPUE) of a given fish species with and without killer whales present. Thus, the full model (not including interactions) used in the analysis can be written as:
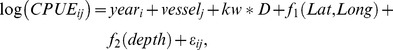
(1)where *CPUE_ij_* is the CPUE of sablefish, halibut, or turbot of a given set by vessel *j* observed in year *i* and *D* is a binary variable that was set to 0 if killer whales are absent and to 1 if they were present. Ninety-five percent confidence intervals were reported as ±1.96 times the standard error. Effect size was calculated as the proportion of deviance uniquely explained by the depredation variable. For each species and area, we fit reduced models that had the same model structure but without the binary depredation variable. The smoothing parameters for the smooth terms (lat/long and depth) in the reduced model were set equal to the values estimated in the full model to maintain comparability. This method provides a minimum estimate of the percentage of deviance uniquely explained by the depredation variable.

### 2.3. Direct costs

#### 2.3.1 Additional fuel costs for the observed longline fleet

**Figure 2:**
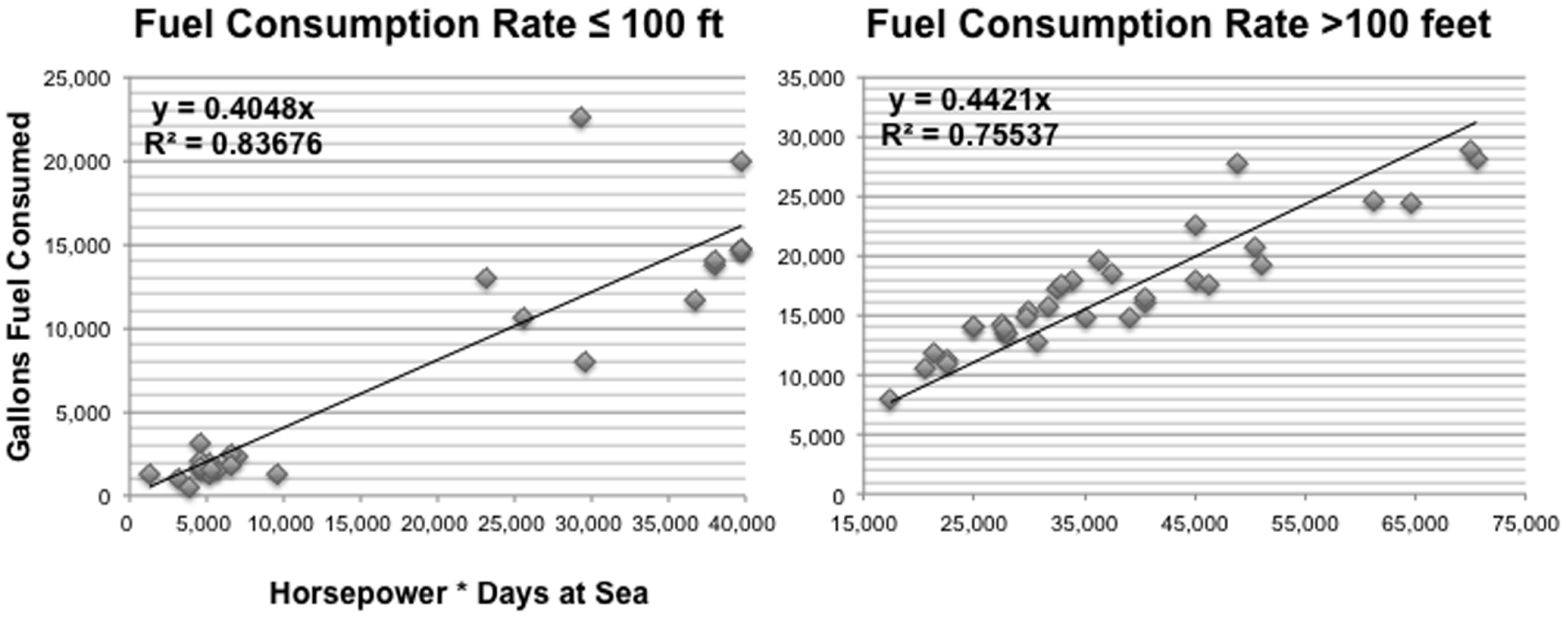
Fuel consumption relationships for longline vessels. These data constitute 60 fishing trips in Alaska in 2011 and 2012 (method from Tyedmers 2001) for vessels less than or equal to 100 ft (A) and vessels greater than 100 ft (B).

Direct costs incurred by the observed longline fleet were determined by estimating additional fuel consumption due to lower CPUEs on killer whale-depredated sets. In these lucrative longline fisheries, fishery participants generally fish until their full quota is caught. Fuel consumption was assumed to increase proportionally to the additional effort required to compensate for diminished CPUEs. Diesel fuel prices per gallon were averaged by year for 1998 using US Energy Information Administration Alaska diesel industrial price data (http://www.eia.gov) and from 1999–2012 using EFIN Fisheries Economic Data Program historic diesel fuel prices for ports in Alaska (http://www.psmfc.org/efin/) [33], [34]. The inflation-adjusted price of marine diesel fuel per gallon increased during the study period from a low of $1.18/gallon in 1998 to a high of $4.35/gallon in 2008; Alaskan diesel fuel prices have remained fairly steady from 2008 through 2013. The total fuel consumption for sablefish, Greenland turbot and Pacific halibut sets was calculated using fishery effort data and a generic rate of fuel consumption for demersal longline vessels in Alaska [35]. Total fuel consumption for observed sets from 1998 to 2012 was estimated separately for vessels ≤100 ft and vessels >100 ft using the following equation:
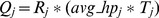
(2)where *Q_j_* is the total quantity of fuel consumed (gallons) for the *j*-th year, *R_j_* is the generic rate of fuel consumption (gallons/(horsepower*sea days), *avg_hp_j_* is the average main engine horsepower for vessels ≤100 ft or vessels >100 ft, and *T_j_* is the total aggregate effort in days at sea for vessels ≤100 ft or vessels >100 ft [35]. In order to determine an average rate of fuel consumption (*R_j_*) to be applied to the observed longline fleet (for which total days at sea and fuel consumption data were not available), days fished and fuel consumption data were collected separately from a select group of longline fishing corporations and individual vessel owners operating in Alaska. In addition to vessel length and horsepower, detailed trip information was provided including: fuel consumed per trip, days fished per trip and days steamed per trip. The rate *R_j_* was estimated by regressing the actual fuel consumed during 26 fishing trips on vessels ≤100 ft and during 34 fishing trips on vessels >100 feet against vessel horsepower times reported days at sea for 2011 and 2012 (Table 1, Figure 2).

**Table 1:** Estimated rate of fuel consumption [gallons/(hp*days)], average engine power (hp), estimated time at sea (days) and estimated fuel consumption (gallons) for observed longline vessels from 1998 to 2012.

Vessel size	R_j_	R_j_ 95% CI	Engine power (hp)	Estimated days at sea	Estimated gallons total	Number of hauls	Avg fuel use per haul (gallons)	Avg fuel use per haul 95% CI (gallons)
**≤100 ft**	0.405	0.359–0.451	633	3401	871,338	4,379	199	±23.1
**>100 ft**	0.442	0.421–0.464	1378	7950	4,843,249	11,357	426.5	±30.0

The total number of sea days for the observed fleet was estimated by inflating the total number of days fished by a constant proportion of days for “steam time.” The steam time to fishing time ratio varied according to vessel size based on results from the 60 fishing trips analyzed. Vessels up to 100 ft used on average 0.5 days of steam time for each day of fishing time [n = 866 days total]; vessels over 100 ft used on average 0.25 days of steam time for each day of fishing time [n = 981 days total]). The average engine power *(avg_hp)* for observed vessels ≤100 ft was 633 hp. For observed vessels >100 ft, the average engine power was 1378 hp. For observed sets impacted by killer whale depredation (n = 819 sets), the amount of additional fuel consumed due to killer whales was estimated by multiplying the average fuel use per set (Table 1) by the model-estimated CPUE differences for each species and management area. The additional fuel used due to killer whale depredation (gallons) was then multiplied by the average price per year of diesel fuel ($/gallon) to obtain an estimate of additional fuel costs. Estimates of additional fuel costs to the observed longline fleet were adjusted for annual inflation rates [36].

#### 2.3.2 Additional direct costs 2011–2012

Average direct costs due to killer whale depredation were also calculated based on information provided by the six longline vessels that collected real-time depredation data on fishing grounds in western Alaska during the 2011 and 2012 fishing seasons. In addition to depredation frequency data, fishermen respondents recorded all depredation avoidance measures they employed including: the use of deterrents, how long they waited if they dropped their gear back down (hrs), how far they steamed if they moved to a different site (nm) and how long they traveled to get to that site (hrs). They also reported estimated gear damage due to straightened hooks and total crew food expenditures for the season. The additional time spent on the fishing grounds (hrs) due to killer whale depredation was calculated by summing the reported additional travel times and wait times (hrs). The additional time spent on the grounds (hrs) was divided by 24 to estimate the total and average additional days fishing vessels were forced to remain on the grounds due to killer whale interactions. Sets where a deterrent was used were not included in the analysis.

The additional cost of food was estimated by multiplying the average cost of food for the crew per day by the number of days each vessel reported extending its trip for a given year. The additional fuel expenditure due to killer whale interactions was estimated as the average fuel consumption (gallons of fuel burned per hour or GPH) multiplied by the additional travel time in a given year as reported by the vessel (hrs) multiplied by the average price ($) of diesel fuel in Alaska for that year [33], [34]. Fuel consumption for each vessel was calculated by multiplying the established specific fuel consumption (sfc) for diesel engines (0.4 lbs per hp) by engine power (hp) of the vessel and dividing the result by the fuel-specific weight (fsw; 7.2 lbs per gallon)[37]. The average inflation-adjusted price-per-gallon of diesel fuel was $3.85 for 2011 and $3.93 for 2012 [33], [36].

### 2.4 Opportunity costs of lost time 2011–2012

The opportunity costs in lost time incurred by the six longline vessels collecting real-time depredation data in 2011 and 2012 in western Alaska were estimated. This approach was based upon traditional time allocation theories linking the opportunity cost of lost time to foregone earnings [38]. This can be extended such that a relevant wage rate can be used as a proxy for the opportunity cost of lost time [39], [40]. In Alaskan sablefish and Pacific halibut IFQ fisheries, crew are generally paid a crew share based on vessel profits, as opposed to the crew receiving a daily wage rate [22]. Thus, if a vessel were forced to stay on the grounds an additional day due to whale interactions, the crew would incur opportunity costs in foregone wages that could have been earned in another occupation that day. Opportunity costs in lost time per vessel were estimated as the average daily income of male workers multiplied by the number of crew per vessel multiplied by the number of additional days each vessel was forced to remain on the fishing grounds due to killer whale depredation. An alternative valuation approach based upon the Travel Cost Method (TCM; generally used in recreation studies) was also considered. TCMs are often used in non-market valuation recreational demand models and typically assume that site visits are valued by out-of-pocket expenses and opportunity time costs of travel to and from a given site [38], [40], [42]. The opportunity cost for the TCM analysis was assumed to be 30% to 60% of the average wage rate, which brackets the likely range [42], [43]. Given the commercial nature of this fishing, however, wages are considered as the appropriate opportunity cost. There may be additional opportunity costs, but this is a reasonable lower bound. US Census data were used to estimate average daily income of male workers by reported vessel homeport city [41].

### 2.5 Ethics statement

The Institutional Review Board at the University of Alaska Fairbanks approved all research involving human subjects under this study (IRB # 221381-2). Fishermen respondents recorded basic seasonal vessel information and daily depredation data, and written consent was obtained for all participants.

## Results

### 3.1 Frequency of killer whale depredation

**Figure 3:**
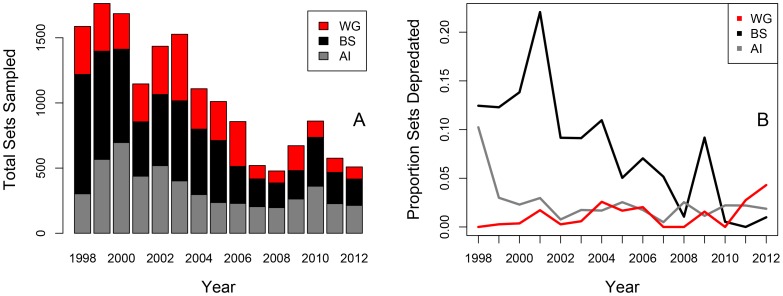
Number of sets sampled and proportion depredated by area. Total number of sets sampled (n = 15,749) in all three management areas (A), and the proportion of sets depredated by killer whales over time by region (B).

A total of 15,749 sets targeting sablefish, Greenland turbot or Pacific halibut were sampled by NMFS on-board observers in the BS, AI and WGOA between 1998 and 2012 (Figure 1). A total of 5.2% of sets were affected by substantial killer whale depredation across all three management areas and species. The highest percentages of sets depredated occurred in the BS for each species (sablefish 21.4%, Greenland turbot 9.9%, Pacific halibut 6.9%; Table 2). The overall number of observed sets declined from 1998 to 2012, and the proportion of sets impacted also declined during the period (Figure 3). Sets targeting Greenland turbot had the highest level of depredation across all management areas combined as measured by the proportion of sets affected (8.9%; Table 2). The estimated proportion of skates affected by killer whale depredation during the NMFS sablefish longline survey [5] was higher than the estimated proportion of sets impacted based on the observer data (this study) (Table 2). From 1998 to 2012, a total of 60,720 skates were sampled on the longline survey in the BS, AI and WGOA, and the percentage of skates depredated by killer whales across all years and areas was 21.7% (Table 2, Figure 1).

**Table 2:** The percentage of sets or skates impacted by killer whales.

	Bering Sea			Aleutian Islands						Western Gulf of Alaska					
	% Affected	(n)		% Affected			(n)			% Affected			(n)	Overall % Affected	
**NMFS Observer Data 1998–2012**															
Sablefish	21.4%	252		2.3%			2614			1.0%			2850	**2.5%**	
Greenland turbot	9.9%	4909		4.5%			963			NA			NA	**8.9%**	
Pacific halibut	6.9%	1577		1.7%			1533			1.2%			1008	**3.6%**	
**NMFS Longline Survey 1998–2011**															
Sablefish	34.6%	19075		9.2%			17102			20.0%			23913	**21.7%**	
**Written survey results (n = 95 respondents)**															
Sablefish/Halibut														**10–25%**	
**Depredation Data Sheets 2011–2012 (n = 846 sets)**															
Sablefish/Turbot/Halibut														**9.6%**	

The percentage of sets (NMFS observer data 1998–2012) or skates (NMFS longline survey data 1998–2012; [5]) affected by killer whale depredation by target species and management area, with sample size included for each item. Results from written surveys [7] and depredation data sheets are also included.

Written surveys and collaborative depredation research with longline fishermen were also used to evaluate the proportion of sets impacted by killer whale depredation. [7]. Six skippers onboard longline vessels completed depredation data sheets on the grounds for fishing days when interactions occurred with killer whales. A total of 81 out of 846 monitored sets (9.6%) were reported as impacted by killer whale depredation throughout the study period from 2011 to 2012, and depredation occurred on 57 days of the 262 days fished (21.8%). The percentage of sets affected differed among vessels, ranging from 4.7% to 15.4% in 2012 (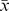 = 9.1%) and from 11.1% to 26.7% (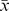 = 18.5%) in 2011. In an earlier study, 95 longline fishermen in Alaska completed written surveys estimating the proportion of sets affect by killer whales [7]. The majority of written survey respondents reported that 10–25% of sets were depredated (Table 2).

### 3.2 Observed fishery CPUE reductions

The estimated reduction in observed fishery CPUE associated with killer whale depredation, averaged across all depredated hauls and accounting for differences among vessels and years as well as for spatial patterns in CPUE, ranged from 35.1% to 69.3% (p<0.0001) among areas and species. The estimated killer whale coefficients were significant for all species in all areas (p<0.0001), with the exception of Pacific halibut in the WGOA (p = 0.45). Residual diagnostics did not indicate autocorrelation between years. The greatest CPUE reduction for depredated sets occurred for Bering Sea sablefish (69%), followed by AI Greenland turbot (67%), and WGOA sablefish (65%; Table 3). When averaged across all management areas, sets dominated by sablefish incurred the greatest CPUE reductions (63%), followed by Greenland turbot (60%) and Pacific halibut (36%; Table 3).

**Table 3:** Model-estimated killer whale depredation coefficients (*1-exp^kw^*), with the percentage of deviance explained (%Dev) by the killer whale depredation coefficient and final model (Equation 1) and sample size (n).

	Reduction CPUE (kw)	95% CI (kw)	p-value (kw)	%Dev (kw)	%Dev (full model)	n	% Sets Affected
**Sablefish**							
**Bering Sea**	69%	58–77%	p<0.0001	10.8%	65.9%	252	21.4%
**Aleutian Islands**	55%	46–62%	p<0.0001	2.3%	25.2%	2614	2.3%
**Western Gulf of Alaska**	65%	56–72%	p<0.0001	2.2%	25.6%	2850	1.0%
**Greenland turbot**							
**Bering Sea**	54%	50–57%	p<0.0001	6.1%	35.6%	4909	9.9%
**Aleutian Islands**	67%	57–74%	p<0.0001	4.8%	40.6%	1006	4.5%
**Pacific halibut**							
**Bering Sea**	35%	21–47%	p<0.0001	0.1%	49.7%	1575	6.9%
**Aleutian Islands**	57%	36–71%	p<0.0001	0.1%	38.9%	1533	1.7%
**Western Gulf of Alaska**	15%	NA	p = 0.45	0.0%	49.9%	1008	1.2%

### 3.3. Costs due to killer whale depredation

#### 3.3.1 Additional fuel costs for the observed longline fleet

The average additional fuel costs per depredated set in the observed longline fleet between 1998 and 2012, as estimated from observer data, was $432.5±$147 (inflation-adjusted). The total time at sea (fishing days + estimated steam time) was approximately 3401 days (2267+0.5*2267) for vessels ≤100 ft and 7950 days (6360+0.25*6360) for vessels >100 ft. Based on these values, the total fuel consumed for all years combined from 1998 to 2012 (*Q_j_*) was 5.7 million gallons ±333,815 gallons (Table 1). The additional fuel costs incurred by individual vessels varied by two orders of magnitude, ranging from $263 to $34,795 (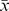 = $6,773). A total of 819 sets were impacted by killer whale depredation during this time, and the inflation-adjusted cost of the additional fuel attributed to killer whale depredation was $358,991±$122,223 for all vessels combined from 1998 to 2012.

**Figure 4:**
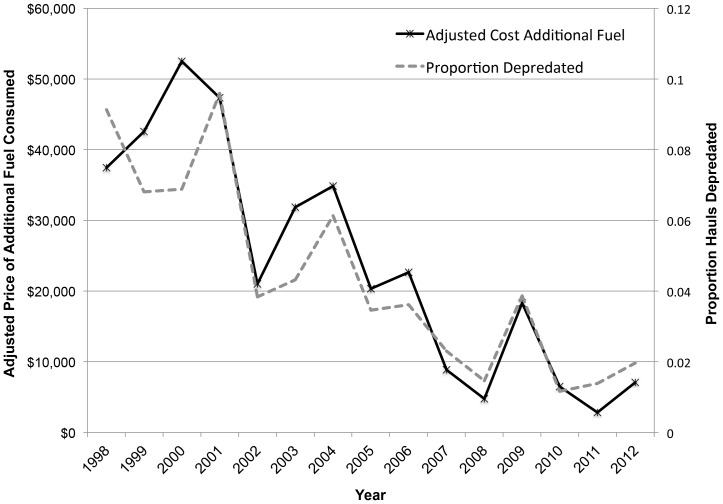
Additional fuel costs and proportion sets depredated. Additional fuel costs incurred by the observed longline fleet due to killer whale depredation and the proportion of sets depredated 1998–2012.

Altogether, changes in Greenland turbot fishing operations accounted for 65% of the increased fuel consumption due to depredation, changes in sablefish fishing accounted for 23% of the increased fuel consumption, and changes in Pacific halibut fishing accounted for 12% of the increased fuel consumption. Fuel cost increases were concentrated in the BS; Greenland turbot operations in the BS alone accounted for 60% of the additional costs incurred due to killer whale depredation for all species in all three management areas. Despite the relatively low number of observed sablefish sets in the BS (n = 252), the consistently high proportion of sablefish sets impacted by killer whales in the BS accounted for approximately 10% of the additional fuel costs. The total costs associated with killer whale depredation declined over time in concert with the proportion of sets depredated (Figure 4). Killer whale depredation accounted for an 82% increase in fuel expenditures to catch the same amount of quota when considering depredated sets only and for a 5% increase in fuel expenditures across depredated and non-depredated sets.

#### 3.3.2 Additional direct costs 2011–2012

Based on data collected by fishermen respondents in 2011 and 2012, the proportion of effort and number of sets affected by killer whale depredation was highest in the WGOA, followed by the BS and the AI. The majority of sets were targeting sablefish in all three areas, however, Greenland turbot sets in the BS and Pacific halibut sets in all three management areas were also included in the analysis. The most fishing days with recorded whale interaction data occurred in May, but killer whale depredation data was recorded as early as April 1 and as late as July 20. The minimum number of killer whales reported interacting with a vessel ranged from 1 to 30 (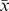 = 6.5) and the maximum number of whales ranged from 2 to 40 (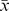 = 12.1). When fishermen were forced to “fish through the whales” (generally due to weather or predation by “sand fleas”), respondents reported an average of 56% of catch was lost to killer whales. On most (93%) of killer whale affected sets, fishermen opted to employ depredation avoidance measures, and this information was recorded. Respondents most frequently reported dropping their gear and waiting to haul (50.0%) followed by moving to a new location (29%). Other reported measures included the use of acoustic or physical deterrents (∼14%). Fishermen opted to fish through the whales on 7% of sets.

Respondent answers to depredation avoidance questions were used to estimate some of the direct costs a vessel experiences when avoiding depredating killer whales. Respondents reported waiting or traveling for a total of 809 hrs or 34 days (495 hrs in 2012, 314 hrs in 2011) due to killer whale depredation. Individual vessel wait times varied from 1–50 hrs (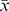 = 17.5 hrs) per set. Respondents reported steaming for a total of 1226 nm (889 nm in 2012, 337 nm 2011) and individual set steam distances by vessel ranged from 4–110 nm (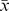 = 36.1 nm). The average additional travel distance per vessel for a given season was approximately 204 nm (Table 4). One of the primary costs incurred by vessels participating in this study was associated with the increased fuel consumption to evade the whales (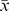
_ = _$4,677 per vessel per season or 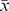 = $ 411 per vessel per depredated day; Table 4). When killer whales interacted with a longline vessel during the study period, the estimated average direct cost of depredation avoidance (based on fuel and crew food) was 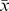 = $5,618 per vessel and 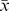 = $494 per depredated day.

**Table 4:** Fishermen collected depredation data.

Additional Distance Traveled	Extended Trip Time (travel and wait time)	Additio-nal Fuel	Additional Crew Food	Opportunity Cost Not Working	Total Additional Expenditures		
Avg per vessel (nm) per season	Avg per vessel (hrs) per season	Avg per vessel per day ($)	Avg per vessel per day ($)	Avg per vessel day ($)	**Total ($)**	**Avg per vessel per season ($)**	**Avg per vessel per day ($)**
204 nm	162 hrs	$411	$83	$309	**$45,685**	**$9,137**	**$802**

Reported additional distances traveled, extended trip times, calculated additional fuel expenses and additional crew food costs, and the estimated opportunity cost of lost time (wage rate) due to depredating killer whales (n = 846 sets).

#### 3.3.3 Opportunity costs of lost time 2011–2012

Fishermen respondents recorded their additional wait and travel time when they avoided killer whales for a total of 809 hrs or 34 days due to killer whale interactions. Vessel wait/travel times resulted in an estimated opportunity cost of $522 per vessel per additional day spent on the grounds (n = 34 days) or a total of $17,596 for all vessels combined in 2011 and 2012 (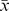 = $309 per depredated vessel-day (n = 57 days; Table 4). Reported wait times per vessel ranged from 75 hrs to 260 hrs (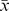 = 162 hrs) per season; however, examining the ratio of days waited to days fished (setting or hauling gear) may be a more relevant comparison and ranged from 0.053 to 0.264 (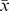 = 0.168).

## Discussion

### 4.1 Frequency of killer whale depredation

This study synthesizes analyses involving multiple data sources to estimate the frequency of killer whale depredation on commercial longline fisheries targeting sablefish, Greenland turbot and Pacific halibut in western Alaska and some of the economic impacts these interactions are having on the fleets. The proportion of observed commercial fishery sets impacted by killer whales was highest in the BS (7–21%), followed by the AI (2–5%; Table 2). Fishermen respondents on the grounds reported that approximately 10% of monitored sets were affected in 2011 and 2012 in all three management areas. These results are fairly consistent across the two commercial fishery data sources used in this study, with the exception of the proportion of sets depredated in the WGOA region for the observed longline fleet (∼1%). The low proportion of observed sets affected by killer whales in the WGOA may be attributable to a number of factors: killer whale depredation is a relatively recent phenomenon in the WGOA region; there are gaps in the spatial distribution of killer whales in the WGOA [4], [5]; or fishermen in the WGOA may be less likely to fish through the whales.

The number of observed sets declined between 1998 and 2012 (Figure 3A), which may represent transitions in the sablefish and Greenland turbot fisheries in the BS. The Total Allowable Catches (TACs) allocated to the Greenland turbot and Pacific halibut fisheries generally declined in BS due to reduced biomass estimates during the study period [22], [22,24]. Sablefish catches have declined in the BS, and Individual Fishing Quotas are typically not harvested to their full extent [25]. In addition, the BS sablefish fishery has recently experienced a shift away from longline gear towards pot gear, and the timing of the longline Greenland turbot fishery in the eastern BS is reportedly shifted to avoid killer whales [5], [22], [44]. Killer whale depredation may have played a role in shaping some of these operational and gear changes in the BS sablefish and Greenland turbot fisheries.

The proportion of depredated sets also declined during the study period (Figure 3B), likely due to a combination of biotic and abiotic factors influencing the frequency of interactions. The decline in depredated sets also could be representative of spatial and temporal avoidance of whales by the fishery. For instance, only 4% of the days vessels fished through whales on three or more sets in one day occurred after 2004 (and only 16% of the days vessels fished through whales on two or more sets in one day occurred after 2004). Lastly, the target species of each set was assigned based on whichever groundfish species was most prevalent in the set, and it is possible that this method may have resulted in a biased estimate of the number of sablefish, halibut, or Greenland turbot sets impacted by killer whales.

The NMFS sablefish longline survey data consistently showed a higher proportion of skates affected by killer whale depredation in each management area (9.2%–34.6%, Table 2). The NMFS survey records depredation at a different scale (per skate or 45 hooks) than the commercial fishery, which tracks depredation per set (thousands of hooks). Although these data are not directly comparable, the lower percentage of commercial sets impacted by killer whales suggests that commercial fishermen actively avoid killer whales and generally will not fish through killer whale depredation. This is supported by the finding that fishermen respondents collecting real-time depredation data on the grounds only chose to fish through the whales on 7% of the sets when whales were encountered. Fishing through the whales was reportedly done out of necessity. Reasons for this included: “sand fleas were terrible,” “last set of the trip,” or “weather approaching.” In contrast, the NMFS survey is required to fish a given station irrespective of the presence of depredating whales to ensure consistent sampling over time. Thus, it is possible that the proportion of sets impacted on the NMFS sablefish survey may be indicative of the proportion of sets that would have been impacted if fishermen had not employed mitigation measures. However, because fishermen target areas with higher concentrations of sablefish, it is not possible to estimate the degree to which depredation would occur without avoidance measures.

### 4.2 CPUE reductions

Estimates of CPUE reductions due to killer whale depredation in this study concur with a previous assessment of catch reductions in Alaskan waters using NMFS sablefish survey data 1998–2011 [5]. Sablefish CPUE was most heavily impacted by killer whale depredation, with reductions ranging from 55%–69%, closely followed by Greenland turbot reductions (54%–67%; Table 3). Pacific halibut CPUE reductions were relatively less severe, averaging 36% across all three areas. In the earlier study, killer whales were shown to selectively target sablefish (54%–72%) and Greenland turbot (72%) in western Alaska [5]. In a separate study using Generalized Linear Models to estimate the killer whale effect on CPUE, killer whales depressed Patagonian toothfish CPUE by as much as 50% around South Georgia [10]. Alternatively, studies have examined catch damaged as opposed to CPUE depression. Comparing catches between depredated and non-depredated sets may be more effective for tropical, hard-billed fish species such as tuna (*Thunnus* spp.) or swordfish (*Xiphias gladius*), where there is often evidence of a hooked fish damaged and left on the fishing gear. Killer whale depredation was associated with 55% catch damage in the Indian Ocean and 12.4% catch damage off Southern Brazil [11], [15]. Sablefish and flatfish such as Greenland turbot and Pacific halibut typically break away from the hook entirely, thus, estimating changes in CPUE is likely the most appropriate method to date for quantifying the killer whale depredation effect on Alaskan demersal fisheries.

### 4.3 Direct costs and opportunity costs

The largest reported component of direct costs incurred by longliners was additional fuel consumption associated with moving to new fishing areas in response to the presence of killer whales or additional fuel consumption associated with fishing for additional days to make up for lower catch rates due to killer whale depredation. The estimated cost of additional fuel used to move to avoid the whales (fishermen respondent data 2011 and 2012; $289 per depredated set) was lower than the estimated cost of additional fuel used to fish through the whales (observer data 1998–2012; vessel average $433±$147 per depredated set). The total average cost to avoid the whales for fishermen respondents in 2011 and 2012 was $564 ($289 fuel, $58 food, $217 opportunity costs) per depredated set. Comparable opportunity costs were not calculated for the observed vessels from 1998 to 2012 due to data limitations.

It is important to note that the fishermen respondent-reported $289 in additional fuel used to avoid the whales does not take into account other direct costs such as food and the opportunity cost of lost time. Conversely, if an observed vessel fished through the whales on multiple sets per day or per trip, they would have to fish more sets to catch their target quota for that trip. Thus, the overall costs per depredated trip could be significantly higher if vessels opted to fish through the whales on multiple sets, especially if opportunity costs associated with longer trips were taken into account. For example, there were 819 observed sets impacted by killer whale depredation over 445 days. Individual vessels fished through the whales between 2–4 sets per day on 40% of the total 445 days, and the maximum additional cost incurred by one vessel that fished three sets on one day was $2449±$805 based on fuel alone.

Other direct costs and opportunity costs associated with killer whale depredation not taken into account in this analysis could add to the depredation costs incurred by the fleet. For instance, extra bait costs associated with lower CPUEs over time could result in substantial direct costs to the fleet. Bait costs were not included in this analysis as bait type (generally herring, squid, or pollock) and usage varies substantially across vessels and fisheries. Nonetheless, tracking bait costs and additional bait used would be a useful component to future depredation-costs research. Furthermore, baiting additional sets to make up for lost catch would take additional time on each killer whale depredated trip, which would lead to increased opportunity costs in lost time. There may be additional opportunity costs for the vessels if in addition to lost time they are forgoing opportunities to fish in other fisheries, but we do not have data on the value or prevalence of these potential opportunities. Reduced product quality due to killer whale interactions could also result in additional depredation costs not considered in this study. Diminished groundfish product quality due to extended gear soak times (e.g., sand fleas, seafloor abrasion) is another potential depredation cost [45]. For instance, sablefish products are “graded” on quality and size, and fish that are torn or damaged are priced lower.

This analysis shows that fishermen often opt to let their gear soak longer so as not to feed depredating killer whales, but with this decision they risk reducing product quality and revenue. Fishermen also incur greater risk of losing their fishing gear with extended soak times, especially in areas like the AI where currents can be strong and extremely variable. Alternatively, if fishermen are forced to fish through the whales, depredation can result in extra costs in gear damage in straightened and/or bent hooks. Depredating killer whales may target grounds with high fish CPUEs [5]. In response, fishermen may choose to fish in areas with lower CPUEs to avoid depredating whales. That is, whales may be effectively closing down certain fishing grounds where the likelihood of whale interactions is perceived to be high. It is possible a fisher location choice model could be implemented to estimate costs associated with fishing in lower CPUE areas[46], [47], but such analyses would require more refined spatial and temporal information on expected whale depredation rates.

A number of the direct cost estimations for the observed longline fleet as part of this study necessitated assumptions or generalizations about fishing behavior and fuel consumption for the observed vessels from 1998 to 2012. In particular, for this analysis we did not have access to the actual total days fished and steamed (total days out) by each vessel. This value was estimated for the observed fleet based on a subset of vessels (constituting 60 longline trips) for which days steaming and fishing data were available. The estimated ratio of steaming time to fishing time from those observations was applied to the observed longline fleet to approximate the total days each vessel spent getting to or fishing on the grounds for vessels up to 100 ft and vessels greater than 100 ft. The estimate of total days per trip was then used to calculate the overall fuel consumption (and additional fuel consumed due to killer whales) for the observed fleet. Both of these methods are subject to many uncertainties that we were unable to fully quantify in this analysis.

Future studies should attempt to quantify or minimize uncertainties by obtaining more precise estimates of important quantities such as the steaming time to fishing time ratio and fuel consumption data. One approach to improving estimates of fuel consumption and other parameters would be to account for heterogeneities within and among vessel categories, for example based on whether or not fish were processed on board. Fuel consumption rates were averaged based on vessel size, but it is likely that some vessels have improved or modified fuel consumption rates that were not reflected in the available data, which could have resulted in an underestimation of fuel consumed during the early part of the study period when fuel consumption was likely higher. Lastly, historic diesel fuel prices were averaged for each year across western Alaska ports. However, fuel prices vary extensively between ports and fluctuate throughout the year. Thus future studies could benefit from improved resolution of fuel prices based on season timing and fishing port. Despite the challenges inherent in working with these datasets, this study's estimated costs represent a thorough and well-supported approach in a data-limited situation.

## Conclusions

In high-value longline fisheries managed under quota systems, such as the Alaska region sablefish, Pacific halibut and Greenland turbot fisheries, there is typically incentive for fishermen to catch their entire quota, even if it takes longer due to killer whale depredation. In a traditional fishery, depredation can result in lower catches by a vessel for a given year. However, under IFQs, fishermen have the option of fishing longer to make up for the depressed CPUE and still catch their entire quota. Thus, the main costs incurred by Alaskan longline fleets are not due to lost catches but are instead associated with depressed CPUEs and an increased time needed to catch the individual quota. The basic issue for fishery operators dealing with killer whale depredation can be simplified to considering the costs and benefits of fishing choices. Fishery operators experiencing whale interactions essentially have two immediate choices: 1) fish through the whales and incur additional bait and fuel costs and potential gear damage, or 2) wait to haul and/or move to another fishing location and incur additional fuel and crew food costs plus opportunity costs in lost time. Findings from this study suggest that the additional fuel costs of depredation avoidance may still be cheaper than fishing through the whales. If a dollar value were assigned to lost catch, this difference in costs would be even more significant. It is important to note, however, that the opportunity cost of lost time associated with avoiding whales or fishing longer through the whales also represents a substantial cost to the fleet. There is also incentive for fishermen to avoid feeding depredating killer whales so as to limit the spread of the learned depredation behavior and to minimize any reinforcement killer whales receive when trying to remove fish from longline gear.

The groundfish observer program has undergone restructuring, and since 2013 regulations mandate partial observer coverage on vessels 40 ft to 60 ft. These modifications to the observer program should generate additional depredation data for smaller vessels in the fishery. With this enhanced opportunity to collect depredation data, it is critical that fishery interaction reporting criteria be standardized and prioritized within the observer program. The substantial costs and depressed CPUEs associated with killer whale depredation provide strong incentive for fishery managers and fishermen to continue depredation research with special attention to depredation mitigation and potential management solutions.
